# Cachexia and efficiency of trifluridine/thymidine phosphorylase inhibitor + bevacizumab in metastatic colorectal cancer

**DOI:** 10.1038/s41598-024-77766-z

**Published:** 2024-10-29

**Authors:** Masatsune Shibutani, Hideki Tanda, Yuki Seki, Shinichiro Kashiwagi, Tsuyoshi Nishiyama, Yasuhiro Fukui, Daiki Imanishi, Hiroaki Kasashima, Tatsunari Fukuoka, Kiyoshi Maeda

**Affiliations:** 1 Department of Gastroenterological Surgery, Osaka Metropolitan University Graduate School of Medicine, 1–4–3 Asahi-machi Abeno-ku, Osaka City, 545-8585 Osaka Prefecture Japan; 2 Department of Breast Surgery, Osaka Metropolitan University Graduate School of Medicine, Osaka, Japan

**Keywords:** Colorectal cancer, Cachexia, CXI, FTD/TPI, Bevacizumab, Gastroenterology, Oncology

## Abstract

In later-line treatment of metastatic colorectal cancer (mCRC), there may be large differences in treatment efficacy depending on cancer cachexia. Recently, the cachexia index (CXI), which was calculated from the skeletal muscle mass index (SMI), serum albumin concentration, and neutrophil-to-lymphocyte ratio, was developed to evaluate cancer cachexia. We retrospectively examined the CXI of 80 patients who were treated with trifluridine/thymidine phosphorylase inhibitor (FTD/TPI) + bevacizumab (Bmab) therapy as a later-line treatment for mCRC, and assessed the impact of cancer cachexia on chemotherapeutic efficacy using CXI. Progression-free and overall survival rates were significantly worse in the low CXI group than in the high CXI group, although there were no marked differences in tumor factors, such as the number of metastatic organs or gene mutations, between the two groups. As the cross-sectional area of the iliopsoas muscle was significantly associated with that of the skeletal muscle, the accuracy of the CXI based on the psoas mass index (P-CXI), which is easier to calculate than the SMI, in predicting treatment outcomes was equivalent to that of the CXI based on the SMI (S-CXI). Cancer cachexia is an important factor related to treatment efficacy in later-line treatments, such as FTD/TPI + Bmab therapy.

## Introduction

Trifluridine/thymidine phosphorylase inhibitor (FTD/TPI) is effective even in patients with metastatic colorectal cancer (mCRC) who are refractory to standard therapies, including fluoropyrimidine-, oxaliplatin-, and irinotecan-based chemotherapy, and is expected to further extend survival times in later-line therapy^1^. In addition, the survival benefit of adding bevacizumab (Bmab) to FTD/TPI has been reported^2–4^.

The chemotherapeutic effect is influenced not only by tumor factors, such as the tumor volume, number of metastatic organs, histological type, and gene mutations, but also by host factors, such as inflammation, immunity, and nutrition. The therapeutic effects of FTD/TPI + B vary depending on the patient. It is possible that the cause is related to the host, such as with cancer cachexia. Cancer cachexia is a multifactorial syndrome defined by persistent loss of skeletal muscle mass that cannot be completely reversed by conventional nutrition support^5^. As cancer cachexia has been reported to be associated with reduced efficacy of chemotherapy^6^, in later-line treatments, where many patients with cancer cachexia or pre-cancer cachexia are included, there may be large differences in treatment efficacy depending on cancer cachexia. However, the diagnostic criteria for cancer cachexia are vague, making objective evaluation difficult.

Recently, the cachexia index (CXI), an index for evaluating cachexia, was developed by Jafri et al.^7^. CXI calculated from the skeletal muscle mass index (SMI), serum albumin concentration, and neutrophil-to-lymphocyte ratio (NLR) can comprehensively evaluate sarcopenia, malnutrition, and systemic inflammation. If the CXI can predict the efficacy of chemotherapy, it may be useful for developing subsequent therapeutic strategies.

This study aimed to assess the impact of cancer cachexia on chemotherapeutic efficacy using the CXI in patients treated with FTD/TPI + Bmab therapy for mCRC.

## Patients and methods

### Patients

This retrospective study included 80 patients who were treated with FTD/TPI + Bmab therapy for mCRC at the Osaka Metropolitan University Hospital between January 2016 and December 2023. All the patients enrolled in this study were refractory or intolerant to fluoropyrimidine, oxaliplatin, and irinotecan. This study was conducted in accordance with the Declaration of Helsinki and approved by the Ethics Committee of Osaka City University (approval number: 2020-026). Written informed consent was obtained from all patients. All patients were given the opportunity to opt out of the study.

## Treatment

Patients were treated with FTD/TPI 35 mg/m^2^ orally twice a day on days 1–5 and 8–12 in a 28-day cycle, with Bmab 5 mg/kg administered intravenously every 2 weeks. Treatment was discontinued because of disease progression or unacceptable toxicity. Response evaluations using computed tomography (CT) were performed every 8–10 weeks according to the Response Evaluation Criteria in Solid Tumors (RECIST) version 1.1^8^.

### Data collection

We retrospectively collected clinical and laboratory data, including computed tomography (CT) findings, from the institution’s electronic medical records. Blood samples were obtained within 1 week before the initiation of FTD/TPI + Bmab therapy, and abdominal CT scans were performed within 1 month before the initiation of FTD/TPI + Bmab therapy.

### Calculation of the CXI

**Fig. 1:**
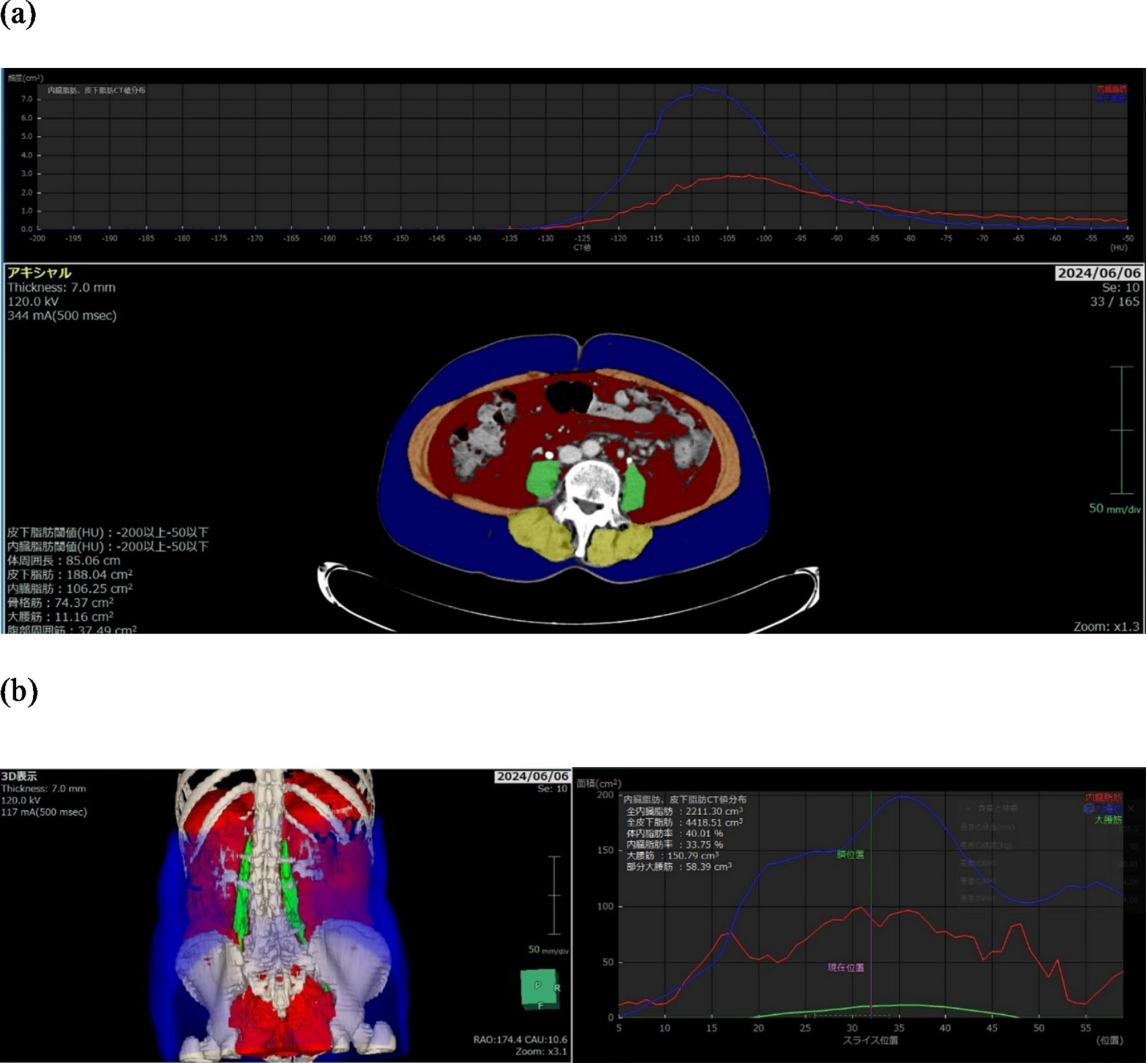
Representative images of body composition components were reconstructed using the SYNAPSE VINCENT 3-dimensional medical image analysis system. (**a**) Cross-sectional computed tomography image at the level of the umbilicus. Areas colored in yellow and orange indicate the skeletal muscle area, and those colored green indicate the psoas muscle area. (**b**) Three-dimensional image construction. Green areas indicate the total volume of the psoas muscle.

The CXI based on the SMI (S-CXI) was calculated as follows: SMI (cm^2^ /m^2^) x serum albumin concentration (g/dL) / NLR. The CXI based on the psoas muscle index (P-CXI) was calculated as follows: The psoas muscle index (PMI [cm^2^ /m^2^]) x serum albumin concentration (g/dL) / NLR. Abdominal CT images taken within 1 month before the initiation of FTD/TPI + Bmab therapy were used to measure the skeletal muscle area (cm^2^) and psoas muscle area (cm^2^). The cross-sectional areas of the skeletal mass and psoas mass were measured at the level of the umbilicus using a 3-dimensional medical image analysis system SYNAPSE VINCENT(Fuji-Film Corporation, Tokyo, Japan) (Fig. 1a). The total volume of the psoas muscle was also measured semi-automatically using SYNAPSE VINCENT (Fig. 1b). SMI (cm^2^ /m^2^) was calculated as the skeletal muscle area divided by the square of height (m^2^). The PMI (cm^2^ /m^2^) was calculated as the psoas muscle area divided by the square of the height (m^2^). The NLR was calculated by dividing the absolute neutrophil count by the absolute lymphocyte count. A receiver operating characteristic curve analysis was performed using the median progression-free survival status to determine the cutoff values of S-CXI and P-CXI separately in male and female patients, considering that muscle mass differs depending on sex.

### Statistical analyses

All statistical analyses were performed using SPSS software package for Windows (SPSS Ver.26; IBM Corp., Armonk, NY, USA). The significance of differences in CXI, clinicopathological factors, and treatment outcomes were analyzed using the chi-squared test, Fisher’s exact test, and Mann-Whitney U-test. The correlation between the cross-sectional area of the iliopsoas muscle at the umbilicus level and other indicators of muscle mass, such as the total volume of the psoas muscle calculated by a 3-dimensional analysis and the cross-sectional area of the skeletal muscle at the umbilicus level, was evaluated using Spearman’s rank correlation coefficient. The overall survival was defined as the interval between the date of initiation of FTD/TPI + Bmab and the date of death from any cause or the last follow-up. Progression-free survival was defined as the interval between the date of initiation of FTD/TPI + Bmab and the date of disease progression, death from any cause, or the last follow-up examination. An objective response was defined as a complete or partial response. Disease control was defined as a complete or partial response or stable disease. Survival curves were estimated using the Kaplan–Meier method, and differences in survival curves were assessed using a log-rank test. Two-sided P values of < 0.05 were considered to indicate statistical significance.

## Results

The study population included 41 men and 39 women, and the median age of the overall population was 70 years (range: 36–88 years). The median follow-up period was 227 days. Seventy-three patients (91.3%) discontinued treatment due to progressive disease, and 3 patients (3.8%) discontinued treatment due to unacceptable adverse events.

The median S-CXI in men and women was 69.76 (range: 5.22–285.63) and 49.62 (range: 17.36–209.61), respectively. The median P-CXI in men and women was 9.87 (range: 0.73–44.70) and 6.69 (range: 0.32–28.18), respectively.

**Fig. 2:**
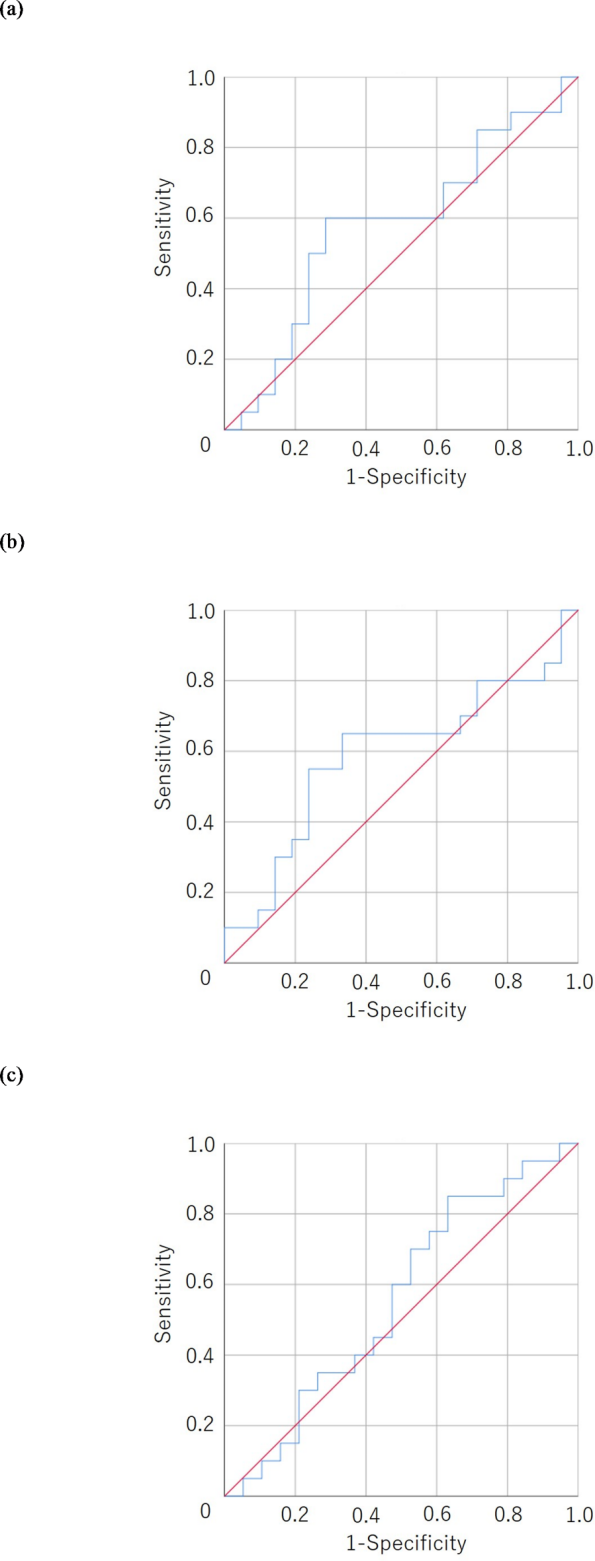

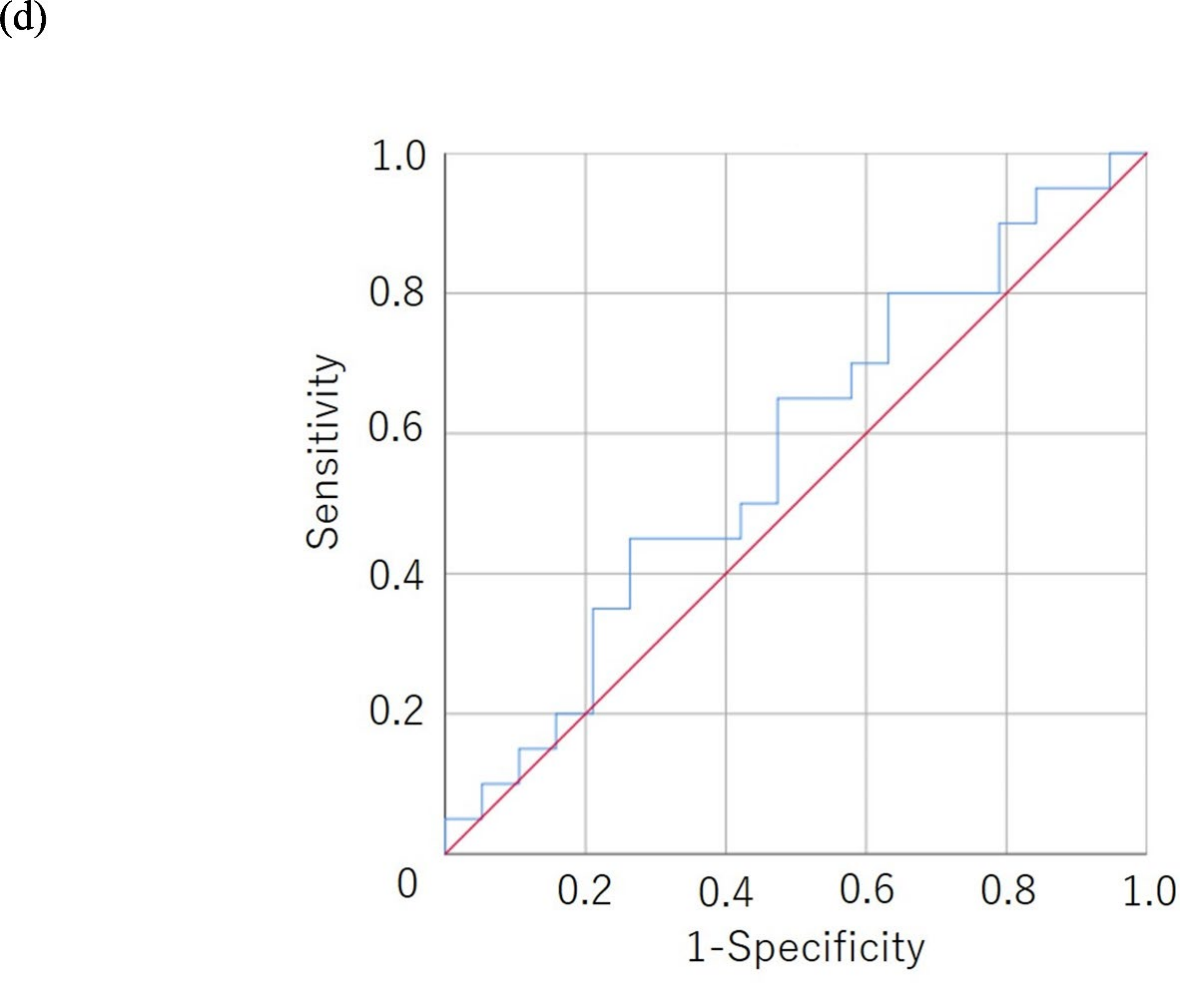
A receiver operating characteristic curve analysis of the cachexia index. (**a**) The cachexia index based on the skeletal muscle mass index (S-CXI) in men. Area under curve = 0.579, 95% confidence interval = 0.398–0.759, *p* = 0.389. (**b**) The cachexia index based on the psoas muscle mass index (P-CXI) in men. Area under curve = 0.590, 95% confidence interval = 0.409–0.772, *p* = 0.322. (**c**) S-CXI in women. Area under curve = 0.555, 95% confidence interval = 0.370–0.740, *p* = 0.535. (**d**) P-CXI in women. Area under curve = 0.574, 95% confidence interval = 0.392–0.756, *p* = 0.431.

The median progression-free survival from the initiation of FTD/TPI + Bmab therapy was 108 days. ROC curve analyses revealed that the cutoff values of S-CXI for men and women were 72.8 and 33.6, respectively, while those of P-CXI for men and women were 9.97 and 5.57 (Fig. 2).

### Associations between S-CXI/P-CXI and clinicopathological factors

The correlations between CXI and clinicopathological factors are shown in Table 1. No correlation was observed between S-CXI/P-CXI and clinicopathological factors, except that low P-CXI tended to be more common in left-sided colorectal cancer.

### Results of a survival analysis according to the S-CXI/P-CXI

**Fig. 3:**
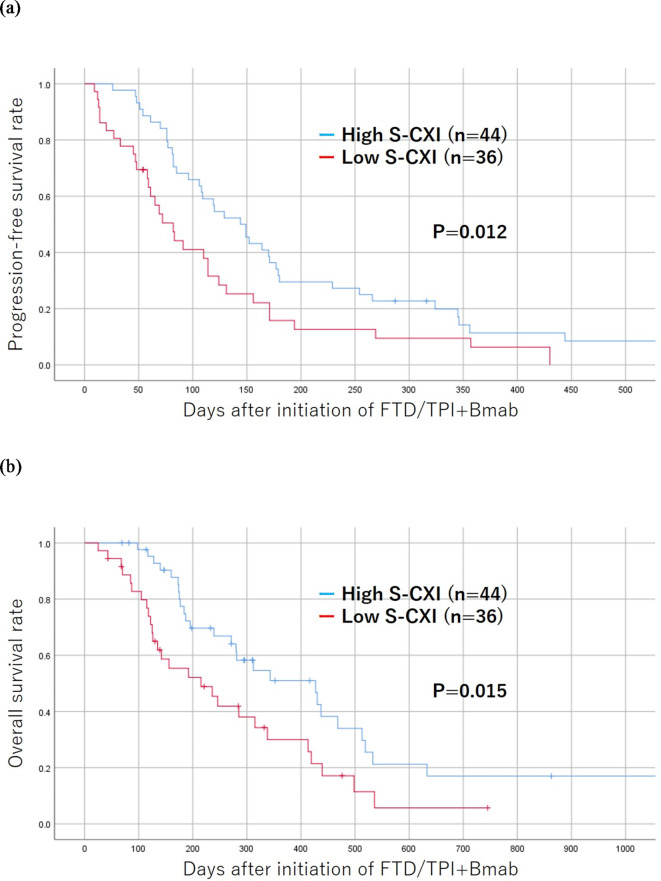
Kaplan-Meier survival curves for the progression-free (**a**) and overall survival (**b**) according to the cachexia index based on the skeletal muscle mass index (S-CXI). The low-S-CXI group showed a poorer prognosis in comparison to the high-S-CXI group with regard to progression-free and overall survival (*p* = 0.012, *p* = 0.015, respectively).

**Fig. 4:**
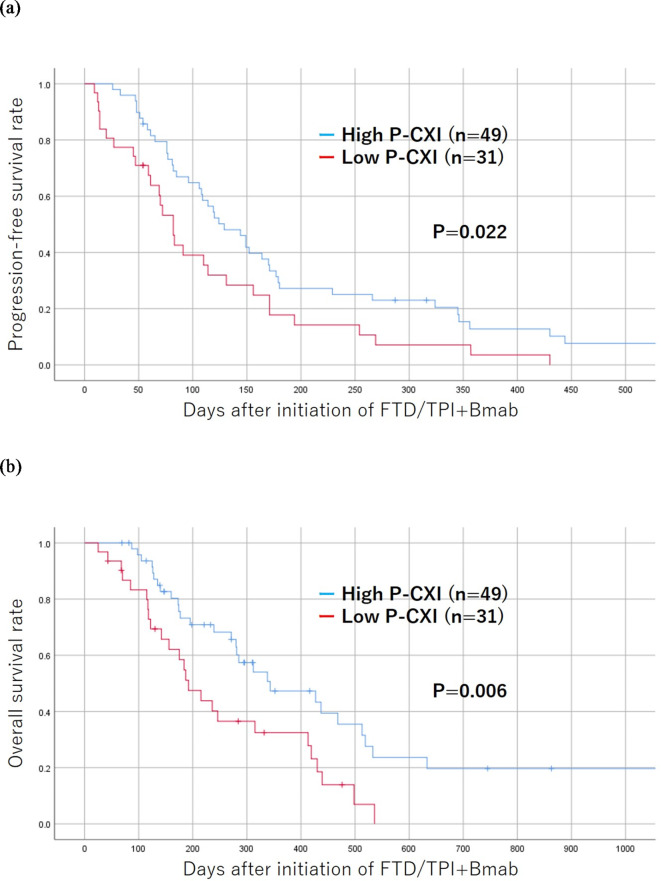
The Kaplan-Meier survival curves for the progression-free (**a**) and overall survival (**b**) according to the cachexia index based on the psoas muscle index (P-CXI). The low-P-CXI group showed a poorer prognosis in comparison to the high-P-CXI group with regard to progression-free and overall survival (*p* = 0.022, *p* = 0.006, respectively).

The progression-free and overall survival rates were significantly worse in the low S-CXI group than in the high S-CXI group (*p* = 0.012 and *p* = 0.015, respectively) (Fig. 3). The progression-free and overall survival rates were significantly worse in the low P-CXI group than in the high P-CXI group (*p* = 0.022 and *p* = 0.006, respectively) (Fig. 4).

#### Correlation between the cross-sectional area of the iliopsoas muscle at the umbilicus level and other indicator of muscle mass

**Fig. 5:**
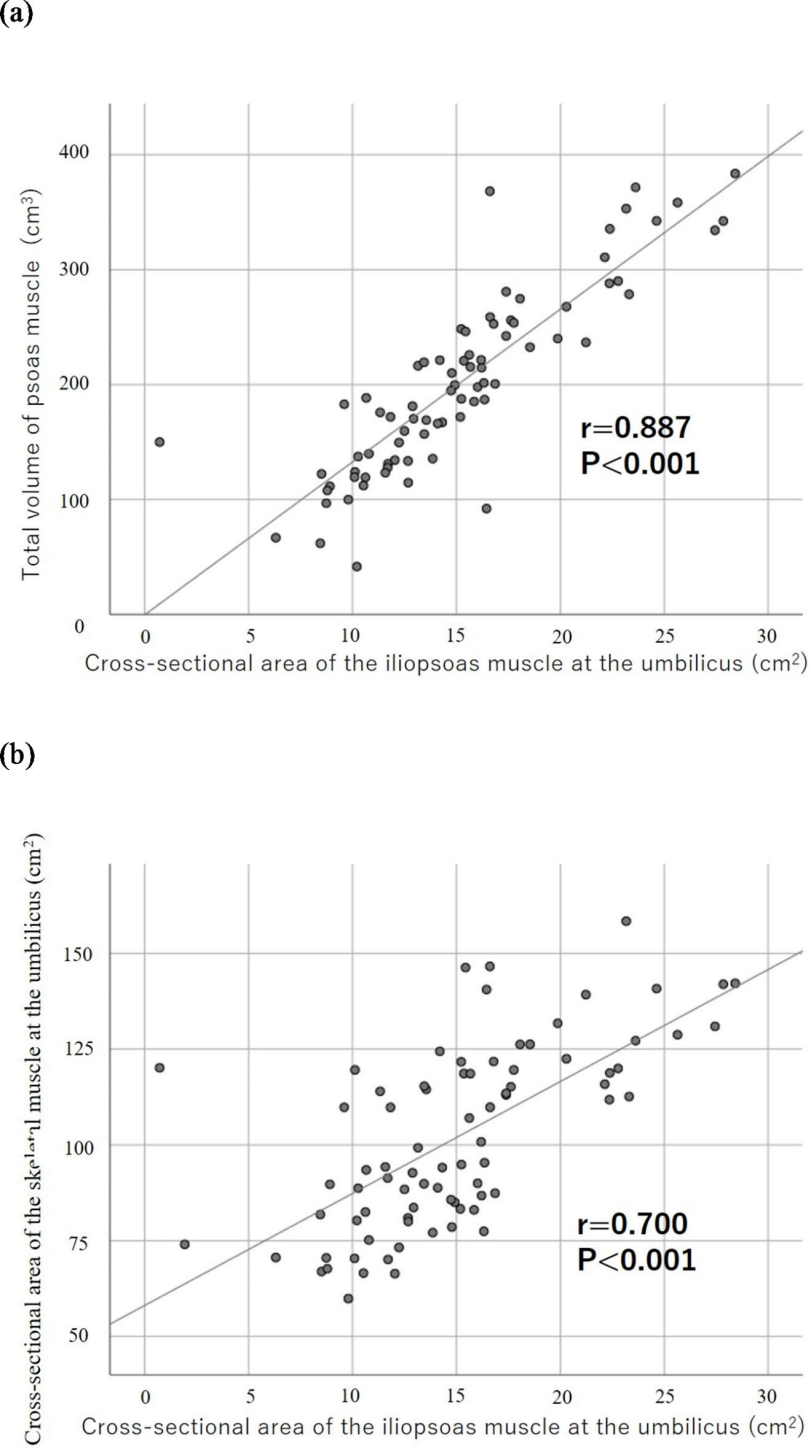
Correlation between the cross-sectional area of the iliopsoas muscle at the level of the umbilicus and other indicator of muscle mass. (**a**) Correlation between the cross-sectional area of the iliopsoas muscle at the level of the umbilicus and the total volume of the psoas muscle calculated by a 3-dimensional analysis. (**b**) Correlation between the cross-sectional area of the iliopsoas muscle at the level of the umbilicus and that of the skeletal muscle at the level of the umbilicus.

The cross-sectional area of the iliopsoas muscle at the umbilicus level was significantly associated with the total volume of the psoas muscle calculated by a 3-dimensional analysis (*r* = 0.887, *p* < 0.001) (Fig. 5a). The cross-sectional area of the iliopsoas muscle at the umbilical level was also significantly associated with that of the skeletal muscle at the umbilical level (*r* = 0.700, *p* < 0.001) (Fig. 5b).

## Discussion

Cancer cachexia has been reported to affect the treatment outcomes in various cancers. For example, CXI has been reported to correlate with the prognosis after curative resection of gastric and colon cancer^9,10^, the efficacy of neoadjuvant chemotherapy for esophageal and gastric cancer^11^, and the efficacy of chemotherapy for unresectable gastric cancer^12^. The incidence of cachexia varies depending on the type of cancer, and it is likely that the appropriate cutoff value for the CXI also varies depending on the type of cancer. However, the fact that cachexia impairs treatment outcomes is common in all cancer types. This study demonstrated that both S-CXI and P-CXI were significantly associated with progression-free and overall survival in patients with mCRC who were treated with FTD/TPI + Bmab therapy as a later-line therapy. Predicting treatment outcomes based on the CXI is useful in daily practice. For patients who are treated with FTD/TPI + Bmab therapy as a later-line therapy and judged to have a poor prognosis based on the CXI, we may need to consider subsequent treatment strategies, including best supportive care.

Cancer cachexia is a multifactorial syndrome characterized by an ongoing loss of skeletal muscle with or without a decrease in fat mass that cannot be fully reversed by conventional nutritional support^5^. Cancer cachexia is caused by anorexia, muscle atrophy, and increased energy consumption due to tumor necrosis factor-α and interleukin-6^9^. Evaluation of the muscle mass, body weight, physical function, and nutritional and inflammatory state are important for the assessment of cancer cachexia. Cancer cachexia reportedly accounts for > 30% of the direct causes of death in cancer patients^13^ and requires careful attention in patients with advanced cancer.

Metabolic changes associated with cancer cachexia may downregulate antitumor immunity^14^. In addition, cancer cachexia increases the adverse events associated with chemotherapy, leading to insufficient doses of chemotherapy^6,15^. Furthermore, myokines released from skeletal muscle and exerting antitumor effects may be reduced in patients with cancer cachexia, because patients with cancer cachexia often have a reduced skeletal muscle mass^16,17^. Therefore, the efficacy of chemotherapy may be lower in patients with cancer cachexia.

Drugs targeting the molecular mechanisms of systemic inflammation, weight loss, and anorexia have been developed. For example, anamorelin, a ghrelin mimetic drug, and enobosam, a selective androgen receptor modulator, have been developed^18,19^. These drugs may improve cancer cachexia and indirectly lead to favorable chemotherapeutic outcomes. In this treatment strategy, the CXI may be useful as an objective indicator to monitor the state of cachexia.

The original method for calculating CXI is based on SMI^7^, but some follow-up reports have calculated CXI based on PMI^9,20,21^. In this study, both CXI based on SMI and CXI based on PMI were associated with the prognosis. The SMI calculation is complicated because there are many measurement points. On the other hand, PMI is relatively easy to calculate because it only requires measurement of the psoas muscle mass. Furthermore, a strong correlation was observed between SMI and PMI, which is consistent with a previous report by Abbas et al.^22^, and PMI is an important indicator of sarcopenia. Therefore, CXI based on PMI is a useful index for clinical application.

In this study, a semi-automatic image analyzer was used to calculate muscle mass, whereas some previous reports have used manually measured long axis × short axis to calculate the psoas muscle mass^21^. Therefore, the results for the cross-sectional area of the muscle obtained in this study were extremely accurate. In previous reports, the skeletal muscle area and psoas muscle area at the third lumbar vertebra, which has been reported to reflect the muscle mass of the whole body^23^, were often used to evaluate the muscle area^10–12^. In contrast, we measured the muscle area at the level of the umbilicus in this study because the image analyzer that we used automatically analyzes muscle mass at the level of the umbilicus. However, because a correlation was observed between the total volume of the psoas muscle calculated by the 3-dimensional analysis and the cross-sectional area of the iliopsoas muscle at the umbilicus level, the cross-sectional area of the iliopsoas muscle at the umbilicus level used in this study may be a valid method for evaluating muscle mass.

The present study was associated with several limitations. This study was principally limited by its small sample size and single-center, retrospective design. In addition, the cutoff value used in this study was a provisional value calculated from the data of the patients who were enrolled in this study. Therefore, large prospective studies should be conducted to confirm our findings and to determine a more accurate cutoff value for CXI as a prognostic marker. Furthermore, the CXI was found to be correlated with the age, and the influence of confounding factors on the chemotherapeutic effect could not be completely excluded.

## Conclusion

Cancer cachexia is an important factor related to treatment efficacy in later-line treatment of mCRC, and CXI is a useful marker for evaluating cachexia.

**Table 1:** Associations between S-CXI/P-CXI and clinicopathological factors.

	S-CXI			*P*-CXI		
Factors	Low (*n* = 36)	High (*n* = 44)	*p*-Value	Low (*n* = 31)	High (*n* = 49)	*p*-Value
Age (years)						
Median (range)	69 (44–83)	72 (36–88)	0.249	53 (44–88)	72 (36–85)	0.288
Performance status, n						
0, 1	33	39		26	46	
2	3	5	0.724	5	3	0.250
Location of primary tumor, n						
Right side	7	16		5	18	
Left side	29	28	0.137	26	31	0.075
RAS status, n						
Wild type	19	18		14	23	
Mutant type	13	26	0.163	15	24	> 0.999
Unknown	4	0		2	2	
Number of metastatic organs, n						
1	14	21		14	21	
≥ 2	22	23	0.500	17	28	> 0.999

S-CXI, cachexia index based on the skeletal muscle mass index; P-CXI, cachexia index based on the psoas muscle index.

## Data Availability

The datasets used and/or analyzed during the current study are available from the corresponding author upon reasonable request.
